# A Case of Chronic Limb Threatening Ischaemia with Severely Impaired Pedal Arteries Successfully Treated with Viscosity Intervention and Subsequent Pedal Bypass Surgery

**DOI:** 10.1016/j.ejvsvf.2026.04.004

**Published:** 2026-04-28

**Authors:** Taro Yamasumi, Keisuke Miyake, Shigeru Miyagawa

**Affiliations:** Department of Cardiovascular Surgery, The University of Osaka Graduate School of Medicine, Osaka, Japan

**Keywords:** Apheresis, Limb ischaemia, Microcirculation, Rheocarna, Rheumatoid arthritis, Viscosity intervention

## Abstract

**Introduction:**

This case report presents a 49 year old man with chronic limb threatening ischaemia (CLTI) and seronegative rheumatoid arthritis who presented with ischaemic foot ulcers, rest pain, and severely impaired pedal circulation. Imaging revealed occlusions of the dorsalis pedis, common plantar, and medial plantar arteries, with no visualised perfusion around the foot wounds, classifying the case as no option CLTI in which conventional revascularisation was deemed unfeasible.

**Report:**

The lateral plantar artery was barely visualised and measured 0.4 mm in diameter, initially considered too small for bypass. As a bridging strategy in an initially deemed no option situation, viscosity intervention using Rheocarna (Kaneka Medix Corporation, Osaka, Japan), a selective apheresis system designed to reduce low density lipoprotein cholesterol and fibrinogen via double plasma filtration, was initiated with the expectation that it would improve rest pain and wound condition, and potentially sufficiently enhance pedal microcirculation to enable bypass surgery. Following this intervention, the patient exhibited improvements in wound size, rest pain, and circulatory status. Notably, ultrasound revealed an increase in diameter of the lateral plantar artery to 0.8 mm, after which bypass to this artery became technically feasible.

**Discussion:**

The combined approach of pre-operative viscosity intervention and subsequent pedal bypass surgery resulted in marked improvement in pedal perfusion and complete wound healing. The patient regained ambulatory function and returned to daily life. This case highlights the potential of viscosity intervention with Rheocarna as an adjunctive pre-operative strategy to convert no option CLTI into operable cases by optimising pedal circulation status.

## HIGHLIGHTS

• A case of no option chronic limb threatening ischaemia (CLTI) was successfully treated with a staged hybrid approach.
• Pre-operative Rheocarna was associated with improved microcirculation.
• Rheocarna selectively removes low density lipoprotein cholesterol and fibrinogen by filtration.
• Improved pedal perfusion enabled a technically challenging but feasible bypass.
• This combined strategy expanded revascularisation options in severe CLTI.

## Introduction

Chronic limb threatening ischaemia (CLTI) is associated with a high risk of major amputation or death, with approximately 20–25% of patients undergoing major amputation within one year if revascularisation is not performed.^1^ While revascularisation remains essential for limb salvage, some patients are classified as “no option” CLTI. These patients lack suitable vessel targets for surgical or endovascular reconstruction due to diffuse arterial disease or microcirculatory impairment, and generally have a poor prognosis.^2^ This clinical situation reveals the need for alternative treatment approaches.

Against this background, Rheocarna (Kaneka Medix Corporation, Osaka, Japan), a selective apheresis system, has been proposed as a potential adjunctive therapy. By improving blood rheology through the reduction of low density lipoprotein cholesterol and fibrinogen via double plasma filtration, Rheocarna has been suggested to enhance microcirculation in patients with CLTI, particularly those initially considered unsuitable candidates for revascularisation or those who responded poorly to previous interventions. Although current evidence is primarily based on single arm studies and lacks validation through controlled trials, these findings indicate a potential therapeutic role for Rheocarna in such challenging cases.^3^, ^4^, ^5^

In patients with advanced arterial disease, the absence of suitable revascularisation targets may result in no option status.^6^ Furthermore, even when revascularisation is technically feasible, profound microcirculatory impairment may limit distal perfusion and lead to poor limb related outcomes.^7^

In the present case, the patient exhibited severe ischaemia with impaired pedal circulation. Imaging revealed extremely narrow plantar arteries and poor angiographic visualisation of the plantar and pedal arteries, classifying the case as no option CLTI. As a bridging strategy, viscosity intervention with Rheocarna was introduced with the expectation that it might improve rest pain, wound size, and pedal circulation, to potentially enable bypass surgery. This approach ultimately led to successful revascularisation and wound healing.

## Case Report

A 49 year old man with a history of seronegative rheumatoid arthritis (SNRA) presented with progressive pain in his right lower leg. Nine years earlier, he had undergone synovectomy of the right ankle, at which time SNRA was diagnosed. Two years before presentation, a skin ulcer developed around his right ankle but resolved spontaneously.

He was a former smoker between the ages of 20 and 34 years and had quit smoking 15 years before presentation. There was no history of diabetes mellitus, hypertension, or dyslipidaemia, he was not on dialysis, and had no family history of cardiovascular disease.

There was no clinical or imaging evidence of chronic venous disease. Skin biopsy at that time ruled out vasculitis. One year before referral, right lower leg pain recurred, and disease flare up was suspected. Immunosuppressive therapy was initiated, and he was referred to the cardiology department at the previous hospital. Written informed consent for publication was obtained from the patient.

**Figure 1:**
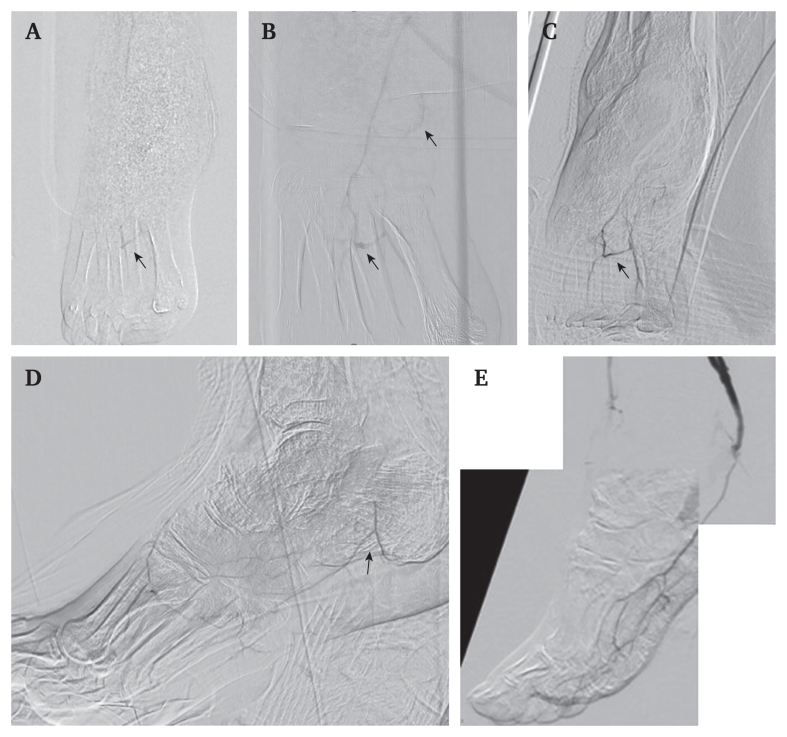
Serial angiographic findings of the pedal arch. (A) Angiography at the previous hospital (anteroposterior view of the lower limb) showed incomplete visualisation of the pedal arch (black arrow indicates the pedal arch). (B) Initial angiography performed at this hospital demonstrated extremely slow retrograde filling of the lateral plantar artery, resulting in a poorly developed pedal arch (black arrows indicate the pedal arch and lateral plantar artery). (C) Following Rheocarna therapy, angiography (anteroposterior view of the lower limb) revealed clear delineation of the pedal arch (black arrow indicates the pedal arch). (D) Following Rheocarna therapy, angiography (lateral view of the lower limb) demonstrated improved visualisation (black arrow indicates the lateral plantar artery). (E) After lateral plantar artery bypass, angiography demonstrated marked improvement with well developed pedal arch circulation.

Angiography performed at the previous hospital confirmed preserved inflow, with no significant stenosis above the popliteal artery, but showed no visualised flow below the ankle, consistent with so called “desert foot”. The patient was informed that major amputation would probably be necessary (Fig. 1A). He subsequently sought a second opinion and was referred to the current department.

**Figure 2:**
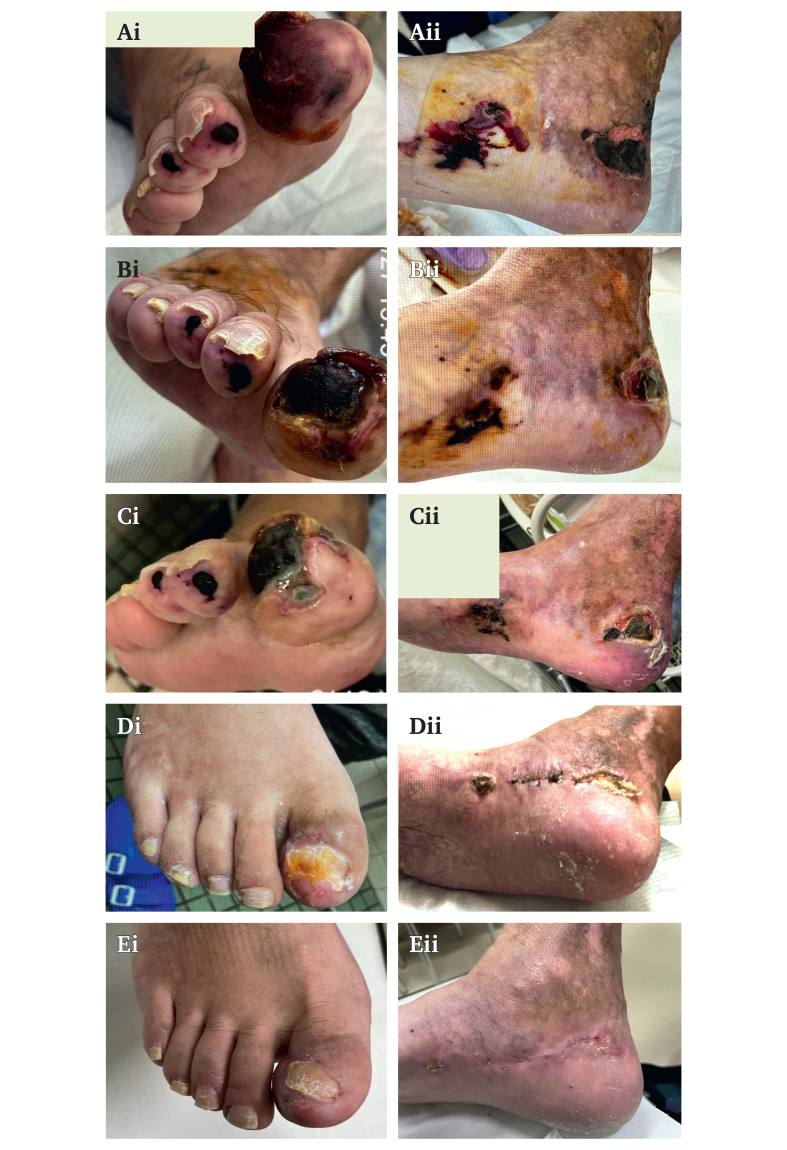
Serial clinical photographs of the same foot illustrating temporal changes in wound appearance during the clinical course. Panels labelled i show the plantar (distal) view of the foot, whereas panels labelled ii show the lateral view of the ankle. (Ai, Aii) On admission, black eschar was present on the right first to third toes and posteroinferior to the medial malleolus. (Bi, Bii) Partial reduction of eschar and ulceration was observed after one month of Rheocarna therapy. (Ci, Cii) Wound size and depth had further decreased after two months of therapy. (Di, Dii) Wound healing had substantially progressed six weeks after the operation. (Ei, Eii) Complete epithelialisation was observed three months after the operation.

**Figure 3:**
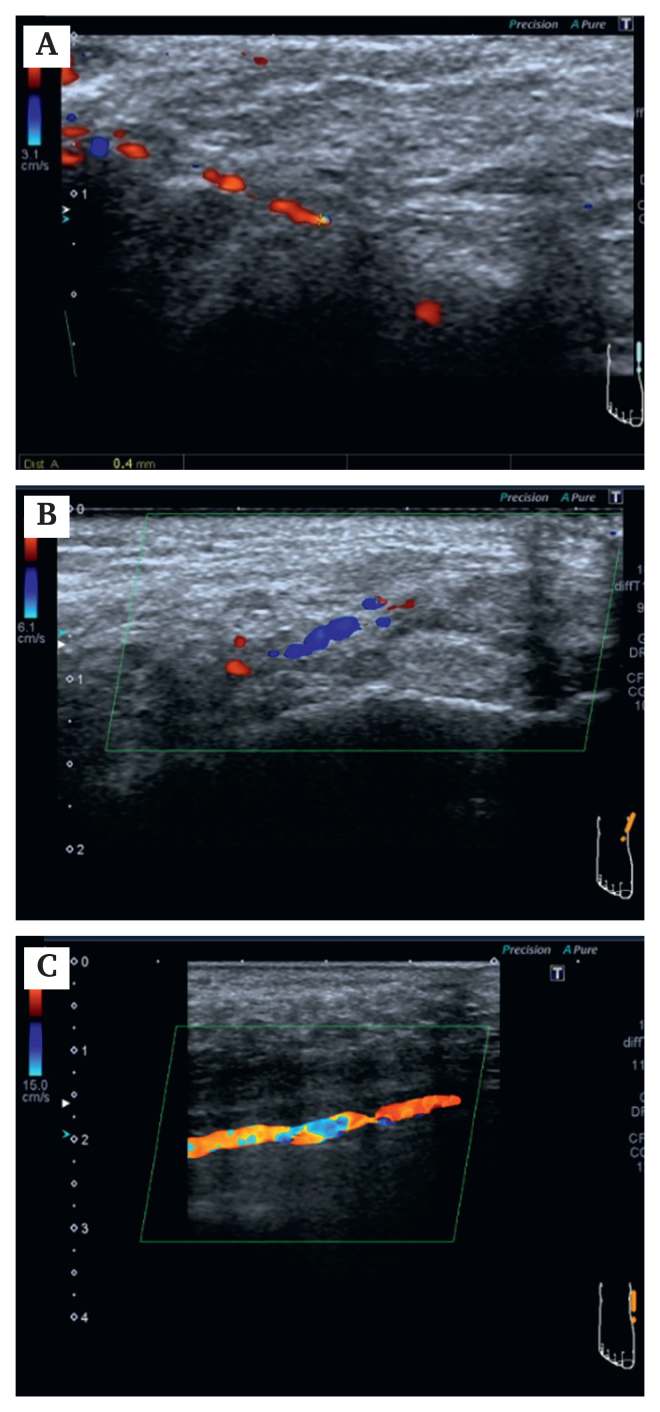
Sonographic images of the lateral plantar artery at (A) baseline, (B) after Rheocarna therapy, and (C) after pedal bypass surgery. Quantitative measurements are provided in Supplementary Table S1. (A) On admission, duplex ultrasound showed severely diminished flow in the lateral plantar artery with a luminal diameter of 0.4 mm. (B) Following low density lipoprotein apheresis therapy, gradual improvement in flow signals was observed, and the artery diameter increased to 0.8 mm after 2.5 months. (C) One month post-operatively, the diameter had further increased to 1.4 mm.

At the time of referral, he was receiving antiplatelet therapy with aspirin, vasodilator therapy, and low dose immunosuppressive therapy with prednisolone (7 mg/day). Ulcers with black eschar were observed on the right first to third toes, postero-inferior to the medial malleolus (4 cm × 4.5 cm), and over the lateral malleolus (Fig. 2Ai, 2Aii). Skin perfusion pressure (SPP) was 12 mmHg on the sole and 10 mmHg on the dorsum, although the ankle brachial index was normal with values of 1.09 (right) and 1.08 (left). SPP measurements were performed under standardised inpatient conditions at controlled room temperature, with the patient in the supine position after adequate rest. In hospital angiography demonstrated extremely slow retrograde filling of the lateral plantar artery from distal branches (Fig. 1B, C). Angiography was performed under identical technical conditions using a 4 F catheter positioned antegradely in the popliteal artery. Pedal perfusion was assessed with undiluted contrast medium (10 mL at 2.5 mL/second) at three frames per second. Subsequent duplex ultrasonography using a 12 MHz linear transducer (Canon Aplio 400; Canon Medical Systems, Japan) identified severely diminished flow in the same segment, with a diameter of 0.4 mm (Fig. 3A).

Rheocarna therapy was initiated in the hope of creating more favourable conditions for possible revascularisation, as bypass surgery was considered technically unfeasible at the time due to multiple critical challenges. Firstly, a large ischaemic ulcer near the medial malleolus was present along the anticipated graft path for lateral plantar artery bypass, raising concerns about graft infection and failure, and necessitating wound size reduction. Secondly, due to extremely poor pedal perfusion, the lateral plantar artery appeared too small for bypass anastomosis.

After 2.5 months of treatment, angiography revealed clear delineation of the pedal arch (Fig. 1D), and the diameter of the lateral plantar artery increased to 0.8 mm (Fig. 3B). At the same time, the ulcer near the medial malleolus decreased in size to 3.5 × 3.5 cm (Fig. 2B, C). The ulcer showed gradual reduction in size and eschar formation during Rheocarna therapy, followed by more rapid improvement after the subsequent bypass surgery (Fig. 2C, D). These changes allowed reconsideration of surgical intervention, as the target artery had become large enough to allow bypass anastomosis, and the reduction in ulcer size enabled an adequate bypass graft route.

The patient underwent a below knee popliteal artery to lateral plantar artery bypass using the ipsilateral great saphenous vein. Intra-operative flow was 8 mL/minute. Post-operative angiography demonstrated a patent bypass graft with improved pedal arch circulation (Fig. 1E). Duplex imaging revealed a patent graft with normal waveform and flow of 7 mL/minute. SPP increased to 73 mmHg (plantar) and 74 mmHg (dorsal). At three months, the lateral plantar artery diameter had increased to 1.7 mm, the graft remained patent with improved limb perfusion, and the wound had completely healed (Fig. 2E; Fig. 3C, D). Serial changes in vessel diameter, skin perfusion pressure, and graft flow are summarised in Supplementary Table S1. Rheocarna therapy was administered only during the pre-operative period and was not continued after surgery. Six months after the operation, the patient had returned to society and was able to walk independently.

## Discussion

This case report presents a challenging case of CLTI successfully treated with bypass surgery following pre-operative Rheocarna therapy. Rheocarna is generally used in patients classified as no option or revascularisation refractory. In this case, the patient was initially considered a no option candidate primarily because of severely impaired pedal perfusion and an extremely small target artery measuring 0.4 mm, which rendered distal bypass technically impractical. Microcirculatory dysfunction represents a major limitation in CLTI, as it adversely affects both wound healing and the durability of revascularisation.^7^ Poor bypass surgery outcomes have been reported in patients with connective tissue diseases or vasculitis associated limb ischaemia, probably reflecting microvascular injury.^8^, ^9^, ^10^ Even partial improvement of microcirculatory function may therefore positively affect revascularisation results and wound healing.

Rheocarna represents a potential adjunctive therapy in this context. In Japan, its use is covered under the national health insurance system for selected CLTI patients with ischaemic ulcers that are refractory to revascularisation or in whom revascularisation is not feasible, and therefore its application is restricted to these specific indications. Under this reimbursement framework, therapy is typically administered twice weekly, with each session lasting approximately two hours, for up to three months. In the authors’ institution, pre-operative use is reserved for carefully selected cases that might otherwise be considered anatomically no option at many centres, such as those with severely compromised pedal runoff or extremely small target vessels. Current evidence supporting Rheocarna remains limited and is primarily based on observational studies. Most published clinical data originate from Japan, where Rheocarna is currently approved and reimbursed, and its broader international availability remains limited. In the TURNIP study, Rheocarna treatment in no option or poor option CLTI patients was associated with clinical improvement.^4^ Importantly, the TURNIP study demonstrated angiographic improvement in approximately 60% of cases, indicating enhanced peripheral perfusion and indirectly suggesting potential benefits for microcirculatory function.^4^ Other studies have reported favourable changes in physiological parameters such as skin perfusion pressure and walking distance; however, these are only indirect indicators of microcirculatory improvement. Reduction of plasma viscosity and fibrinogen levels may decrease microvascular resistance and increase distal perfusion. In this case, increased blood flow through existing collateral pathways may explain the enhanced visualisation and enlargement of the lateral plantar artery, rather than *de novo* collateral formation. These findings may reflect functional haemodynamic changes rather than structural modification. The angiographic pattern showing progressive filling of the pedal arch with visualisation of the terminal lateral plantar artery supports this interpretation. Importantly, surgical feasibility in distal bypass depends not only on vessel visibility but also on sufficient flow and lumen definition to allow safe arteriotomy and anastomosis.

Rheocarna was administered pre-operatively as a bridging strategy. This approach may have improved the microcirculatory environment and increased flow through pre-existing pedal vessels, thereby allowing reconsideration of surgical revascularisation. Ultimately, distal bypass became technically feasible. Although spontaneous collateral formation cannot be completely excluded, the absence of previous clinical improvement and the observed temporal sequence make a purely incidental explanation less likely.

Rheocarna may therefore represent a potential therapeutic option when microcirculatory impairment poses a critical barrier to revascularisation. Future prospective investigations are required to clarify the role of Rheocarna in patients with CLTI, and ongoing multicentre data collection may help to inform appropriate patient selection.

### Conclusion

This case illustrates a potential role of Rheocarna as an adjunctive strategy in highly selected CLTI cases that might otherwise be considered anatomically no option. Pre-operative haemorheological intervention was associated with improved distal perfusion and subsequent technical distal bypass feasibility. However, current evidence remains limited and further studies are required to clarify its clinical role.

## Conflict of Interest and Funding

None.
