# Supplementary material for: A Case of Chronic Limb Threatening Ischaemia with Severely Impaired Pedal Arteries Successfully Treated with Viscosity Intervention and Subsequent Pedal Bypass Surgery
Source: EJVES Vasc Forum. 2026 Apr 28;65:212–7. doi: 10.1016/j.ejvsvf.2026.04.004 (PMC13223980; doi:10.1016/j.ejvsvf.2026.04.004)
Supplement: Multimedia component 1 [file mmc1.docx]

**Supplementary Table S1. Serial changes in lateral plantar artery diameter, skin perfusion pressure, and graft flow.**

| Parameter | Before Rheocarna | After Rheocarna (2.5 months) | Post-operative (1 month) | Post-operative (3 months) |
| --- | --- | --- | --- | --- |
| Lateral plantar artery diameter | 0.4 mm | 0.8 mm | 1.4 mm | 1.7 mm |
| SPP (plantar) | 12 mmHg | 34 mmHg | 73 mmHg | 75 mmHg |
| SPP (dorsal) | 10 mmHg | 31 mmHg | 74 mmHg | 76 mmHg |
| Graft flow (duplex) | — | — | 50 mL/min | 50 mL/min |

SPP: skin perfusion pressure
